# Attitudes Toward Payment for Research Participation: Results from a U.S. Survey of People Living with HIV

**DOI:** 10.1007/s10461-022-03660-2

**Published:** 2022-04-07

**Authors:** Andrea N. Polonijo, Karine Dubé, Jerome T. Galea, Karah Yeona Greene, Jeff Taylor, Christopher Christensen, Brandon Brown

**Affiliations:** 1 Department of Sociology and the Health Sciences Research Institute, University of California, Merced, 5200 North Lake Road, Merced, CA 95343 USA; 2 Gillings School of Global Public Health, University of North Carolina at Chapel Hill, Chapel Hill, NC USA; 3 School of Social Work, University of South Florida, Tampa, FL USA; 4 College of Public Health, University of South Florida, Tampa, FL USA; 5 Department of Global Health and Social Medicine, Harvard University, Boston, MA USA; 6 HIV+Aging Research Project–Palm Springs, Palm Springs, CA USA; 7 Department of Social Medicine, Population and Public Health, University of California, Riverside, Riverside, CA USA

**Keywords:** Incentives, Payment, Ethics, Research participation, HIV

## Abstract

Little is known about how payment affects individuals' decisions to participate in HIV research. Using data from a U.S. survey of people living with HIV (*N* = 292), we examined potential research participants’ attitudes toward payment, perceived study risk based on payment amount, and preferred payment forms, and how these factors vary by sociodemographic characteristics. Most respondents agreed people should be paid for HIV research participation (96%) and said payment would shape their research participation decisions (80%). Men, less formally educated individuals, and members of some minoritized racial-ethnic groups were less likely to be willing to participate in research without payment. Higher payment was associated with higher perceived study risks, while preferences for form of payment varied by age, gender, education, race-ethnicity, and census region of residence. Findings suggest payment may influence prospective research participants’ risk–benefit calculus and participation, and that a one-size-fits-all approach to payment could differentially influence participation among distinct sociodemographic groups.

## Resumen

Se sabe poco acerca de cómo el pago afecta a la decisión de participar en investigaciones sobre VIH. Utilizando data de una encuesta de Estados Unidos sobre personas viviendo con VIH (N = 292), se examinó la actitud de potenciales participantes de investigación acerca del pago, el riesgo de estudio percibido basado en la cantidad del pago, las formas de pago preferidas y cómo estos factores varían según las características sociodemográficas. La mayoría de los que respondieron la encuesta estuvieron de acuerdo en que las personas debían recibir un pago por participar en una investigación sobre VIH (96%) y dijeron que el pago podría moldear su decisión de participar en la investigación (80%). Los hombres, individuos con menor educación formal y los miembros de algún grupo racial-étnico minoritario se mostraron menos propensos a participar en una investigación sin recibir pago. Se asoció un pago más alto con una mayor percepción de riesgos en el estudio, mientras que las preferencias por las formas de pago variaron según la edad, el género, la educación, la raza-etnicidad y la región. Los resultados sugirieron que el pago podría influir en el cálculo del riesgo-beneficio y en la participación de los potenciales participantes en una investigación prospectiva, y que un enfoque único para el pago podría influir de manera diferencial entre los distintos grupos sociodemográficos en lo que respecta a su participación.

## Introduction

Providing payments to research volunteers in exchange for participation is a widely accepted and common practice, including in HIV research where participants often face more than minimal risk [1–3]. These payments, which include money or gifts, are often essential to support participation in research, particularly for higher-risk studies that offer limited direct benefits to participants [3, 4]. While we know payments may be essential to incentivize and offset the cost of participation in research, surprisingly little is known about the factors that researchers and institutional review boards (IRBs) consider important when deciding appropriate payment levels, or how payment amounts affect participants' decisions to participate in research studies. Furthermore, it is unclear how payments may differentially shape prospective research participants’ decisions to take part in studies based on key aspects of their sociodemographic backgrounds, such as their age, gender, race, ethnicity, or socioeconomic status.

Ethical guidelines stipulate that research payments should compensate for time, inconvenience, and burdens but should *not* be considered a benefit of research participation to compensate for risk [5, 6]. Research participants may nonetheless perceive payments as a benefit of research participation. One major ethical concern is that payments could create undue influence to participate in clinical research [7–9]; however, recently scholars have extended this concept to include the potential for underpayment and exploitation of research participants [10–12]. Consequently, identifying appropriate payment amounts for studies that strike a balance between incentivization and ethical payment without being unduly influential can be difficult to achieve.

Scholars have argued for the critical importance of exploring payment decisions in research, as well as the impact of payments on individual research projects [1, 12–15]. For example, Gelinas and colleagues provided a practical framework to rationalize payments and guide IRBs in evaluating their acceptability [13]. The practical framework can be divided into three parts: reimbursements for out-of-pocket expenses, compensation for time and burdens associated with participation in research, and incentives to encourage participation [13]. While a framework can be useful for generating concepts, limited empirical data exist on payments provided in clinical research, and payments are not systematically tracked to permit comparison and reference between studies [1, 16]. Further, most ethics committees do not have any published policies on payment practices, and research payment practices are often inconsistent and highly variable [3, 13, 16, 17].

Research risks, burdens, and benefits may differ significantly between study types and procedures, and these in turn may affect payments (though documentation of this variation is limited). A 2002 review revealed that fewer than one-fifth of U.S. institutions knew which of their studies provided payments [18], while one of the most recent comprehensive studies of payments in U.S. clinical studies (published in 2005) described 467 publications among which fewer than 25% reported payment amounts [3]. Practices and regulations surrounding payment may also vary based on study location. For example, to regulate payments in research, South Africa developed standardized practices [19], while Brazil prohibited payments for clinical research [20]. Outside of these two cases, the absence of reference points for determining appropriate payments often leads researchers to determine payment amounts on a case-by-case basis or contingent on research budgets [18]. Additional factors such as risks and benefits, study procedures, time commitment, historical precedent, IRB recommendations, and local regulators may also affect payment amounts [3, 7, 13, 16, 21].

Before we can define appropriate payment for research participation, we need to assess how different stakeholders view and consider these payments. Among the most important stakeholders in HIV research are the patients/participants themselves, as they ultimately bear the risk and burdens of research participation. Given its commitment to social justice and advocacy and its history of research participation [22], the HIV research community provides an ideal setting in which to better understand the influence of payments on decisions to participate in research, and how such decisions may vary based on participants’ sociodemographic characteristics. Considering the ethical implications of payment for participation in HIV research may be particularly important given people living with HIV are often racial-ethnic minorities, sexual and/or gender minorities, and of lower socioeconomic status, making them vulnerable to exploitation [23–25].

Our study aims to understand how payments affect research participation decisions by asking people living with HIV about their (a) attitudes toward payment for research participation, (b) perceptions of study risk based on payment amount, and (c) evaluations of the relative importance of various forms of payment for research participation. Moreover, we consider whether these attitudes, perceptions, and evaluations vary by key sociodemographic characteristics, including age, gender, education level, employment status, race and ethnicity, geographic region, and self-rated health. Exploring such sociodemographic variation is a critical step toward achieving greater equity in HIV research participation, as the underrepresentation of women, people of color, and other minoritized groups in HIV research contributes to knowledge deficits, insufficient and delayed safety data for new treatments, and disparities in HIV survival [26, 27].

## Methods

### Survey and Participants

We conducted a cross-sectional, internet-based survey among English-speaking adults (aged 18 + years) living in the United States who self-identified as living with HIV. We used Amazon Mechanical Turk (MTurk) for recruitment, a commonly used social science research tool for recruiting populations of interest and collecting rigorous data, comparable to popular U.S. online survey panels [28, 29]. To facilitate high-quality responses, the survey was advertised only to U.S.-based MTurk workers who had successfully completed 1,000 previous human intelligence tasks with a 95% approval rating.

The survey took less than 20 min to complete and was administered in Qualtrics (Provo, UT). Participants provided informed consent by clicking a box stating that they agreed to participate in the survey. They then completed a captcha (as a bot control step) to confirm they were human and were given several screening questions to (a) check for English comprehension and attention and (b) verify HIV seropositive status. English comprehension and attention questions included describing a displayed picture, spelling a word backwards, and selecting a multiple-choice response when directed. Five questions were used to verify HIV seropositive status, including: self-reported HIV status, whether the respondent takes pre-exposure prophylaxis (PrEP; since people diagnosed with HIV would not be prescribed PrEP), HIV medications taken, tests taken to confirm HIV diagnosis, and how HIV is transmitted. Upon survey completion, respondents received a code to submit to MTurk and claim $7 compensation for their time. Given online data collection can be vulnerable to fraud, we carefully reviewed the GeoIP addresses of respondents who completed the survey and rejected those who had screened out before making repeated attempts to qualify [30, 31].

The University of California, Riverside IRB reviewed and approved the study on December 10, 2020 (protocol #HS-20-248). All respondents were recruited, provided informed consent, and completed the survey between March 29 and April 28, 2021.

### Measures

#### Attitudes Toward Payment in HIV Research

The survey assessed respondents’ attitudes toward payment in HIV research using the following questions: (1) “Do you consider payment to be a benefit of participating in research?”, (2) “Would payment play a role in your decision to participate in HIV research?”, (3) “Should people receive payment to participate in HIV research?”, (4) “Should there be any standards or policies on participant payment in HIV research?”, and (5) “If an HIV study did not pay you, would you expect to receive another benefit from participation?”. We constructed dummy (no/yes) variables for each of these questions.

#### Willingness to Participate in HIV Research Without Payment

A categorical variable (coded no [the reference category]/yes/it depends) measured whether respondents stated they would participate in HIV research without any payment.

#### Risk Perceptions

We used a dummy (no/yes) variable to assess whether respondents could imagine a certain risk level from participating in an HIV intervention study in which no amount of payment could convince them to participate. Two continuous variables (ranging from 0 to 100) assessed the (a) percent chance of harm and (b) percent chance of death that would deter respondents from participating in HIV research. The level of perceived risk associated with different study payment amounts was assessed with the question: “If one early-phase HIV intervention study pays you a total of $20,000, and another early-phase HIV intervention study does not pay you, how would you rate the risk of each study?” Respondents indicated the level of risk they rated each study on a scale of 0 (“no risk”) to 10 (“highest risk”).

#### Importance of Various Forms of Payment

Respondents rated, on a scale of 0 (“least important”) to 10 (“most important”), how important each of the following types of payment for research participation are: (a) a cash incentive, (b) reimbursement for lost wages, (c) compensation for time, (d) transportation voucher, (e) food, (f) gifts, and (g) post-trial access to the intervention if proven effective.

#### Sociodemographic Variables

We used binary variables to measure respondents’ gender (man vs. woman), education (no bachelor’s degree vs. bachelor’s degree or higher), and employment status (works full time vs. does not work full time). A categorical variable assessed respondents’ census region of residence: West, Midwest, Northeast, or South (the reference category). Age was measured continuously, in years. Self-rated health was assessed using a visual analogue scale that asked respondents to indicate “how good or bad your own health state is today,” measured continuously from 0 (“worst health you can imagine”) to 100 (“best health you can imagine”).

Race and ethnicity were measured categorically with a single variable, constructed from two questions asking whether the respondent self-identified as (a) Hispanic or Latina/o/x and (b) belonging to one or more of the following groups: White; Black or African American; Asian; Native Hawaiian or other Pacific Islander; American Indian or Alaska Native; or other group. To achieve sufficient power for analyses, race and ethnicity were recoded into a three-category variable as (1) non-Hispanic White (the reference category), (2) non-Hispanic Black, and (3) Hispanic or other/multiple races and ethnicities (hereafter referred to as “Hispanic/other”).

### Statistical Analyses

We calculated descriptive statistics (i.e., frequencies, percentages, means, standard deviations, and ranges) for all variables. We used a series of multivariate logistic regression models to test for significant associations between all sociodemographic variables and respondents’ (a) attitudes toward payment in HIV research and (b) ability to imagine a level of risk that would deter research participation. A single multinomial logistic regression model assessed sociodemographic differences in willingness to participate in HIV research without payment. A series of multivariate linear regression models tested for significant associations between all sociodemographic characteristics, and: (a) percent harm or death that would deter HIV research participation, (b) the perceived risk attributed to studies paying $0 versus $20,000, and (c) the importance of various forms of payment.

We conducted all analyses using Stata SE v. 17 (College Station, TX). We report odds ratios (ORs) for logistic regression models, relative risks (RRs) for multinomial logistic regression models, and unstandardized regression coefficients (*b*s) for linear models. For all models, we report *p* values < 0.05 as statistically significant.

## Results

### Sample

In total, 506 individuals completed the survey and provided a valid code to claim compensation. We rejected 206 responses from individuals who had completed the survey but failed the English language verification questions (*n* = 89) and/or had made multiple attempts to qualify for the survey after screening out (*n* = 117). We accepted responses from the remaining 300 respondents. Our final analytic sample included data from 292 of the 300 accepted respondents who had complete data for all variables of interest (97.33% of the total sample).

Table 1 details descriptive statistics for the sample. Respondents were 18–65 years old (mean = 34.36 years) and had a mean self-rated health of 65.92 out of 100. The majority were male (65.75%), had a bachelor’s degree or higher level of education (72.95%), and were employed full time (82.88%). Over one-third identified as part of a minoritized racial or ethnic group (34.93%).

**Table 1:** Descriptive statistics for all study variables, *N* = 292

Variable	*n* (%)	Mean (SD), range
*Attitudes toward payment in HIV research*		
Considers payment a benefit to HIV research participation	267 (91.44)	
Payment plays a role in their decision to participate in HIV research	243 (80.14)	
Believes people should receive payment to participate in HIV research	279 (95.55)	
Believes there should be standards/policies for payment in HIV research	203 (69.52)	
Expects another benefit from HIV research if not paid	188 (64.38)	
*Willing to participate in HIV research without payment*		
No	111 (38.01)	
Yes	99 (33.90)	
It depends	82 (28.33)	
*Risk perceptions*		
Can imagine a risk level high enough to deter participation	134 (45.89)	
% Chance of harm that would prevent research participation		45.16 (29.67), 0–100
% Chance of death that would prevent research participation		30.66 (32.79), 0–100
Perceived risk rating of study that pays $20,000		6.40 (2.87), 0–10
Perceived risk rating of study that pays $0		2.86 (3.18), 0–10
*Importance of various forms of payment*		
Cash incentive		7.42 (2.90), 0–10
Reimbursement for lost wages		6.55 (3.21), 0–10
Compensation for time		7.82 (2.50), 0–10
Transportation voucher		4.78 (3.34), 0–10
Food		4.22 (3.24), 0–10
Gifts		2.77 (3.07), 0–10
Post-trial access to intervention if proven effective		6.78 (3.17). 0–10
*Sociodemographic variables*		
Age		34.36 (8.71), 18–65
Gender		
Man	192 (65.75)	
Woman	100 (34.25)	
Education		
Less than bachelor’s degree	79 (27.05)	
Bachelor’s degree or higher	213 (72.95)	
Race-ethnicity		
Non-Hispanic White	190 (65.07)	
Non-Hispanic Black	52 (17.81)	
Hispanic/other	50 (17.12)	
Employment		
Not employed full time	50 (17.12)	
Employed full time	242 (82.88)	
Census region		
South	123 (42.12)	
Midwest	43 (14.73)	
Northeast	60 (20.55)	
West	66 (22.60)	
Self-rated health		65.92 (18.29), 10–100

### Attitudes Toward Payment in HIV Research

Overwhelmingly, respondents considered payment a benefit of research participation (91.44%) and believed people should receive payment for participating in HIV research (95.55%; see Table 1). Most stated that payment would influence their decision to participate in HIV research (80.14%), felt there should be standards or policies on payment in research (69.52%), and would expect another benefit from research participation if not paid (64.38%). Table 2 (Models 1–5) shows the results of multivariate logistic regression models assessing differences in each attitude by sociodemographic characteristics. Women were less likely than men to expect other benefits from research participation if not paid (OR = 0.586, *p* < 0.05), controlling for age, race-ethnicity, education, employment, census region, and self-rated health. Significant differences by sociodemographic characteristics were not observed for any of the other attitudes.

**Table 2:** Logistic regression results for attitudes toward payment in HIV research studies (Models 1–5) and multinomial logistic regression results for willingness to participate in HIV research studies without payment (Model 6), by sociodemographic characteristics, *N* = 292

	Model 1		Model 2		Model 3		Model 4		Model 5		Model 6			
	Considers payment a benefit to research participation		Payment plays role in decision to participate in HIV research		People should receive payment to participate in HIV research		There should be standards/policies for payment		Expects another benefit from research if not paid		Willing to participate in HIV research studies without payment (baseline = “no”)			
											“It Depends”		“Yes”	
	OR	CI	OR	CI	OR	CI	OR	CI	OR	CI	RR	CI	RR	CI
Age	.988	[.935, 1.044]	.985	[.951, 1.020]	.964	[.902, 1.030]	1.009	[.979, 1.041]	.987	[.959, 1.016]	1.006	[.971, 1.043]	1.024	[.989, 1.060]
Woman^a^	.592	[.248, 1.411]	.569†	[.307, 1.052]	.442	[.137, 1.419]	1.070	[.618, 1.855]	.586*	[.347, .989]	.987	[.505, 1.927]	2.045*	[1.111, 3.764]
Race-ethnicity^b^														
Non-Hispanic Black	1.036	[.315, 3.409]	1.098	[.487, 2.478]	.910	[.176, 4.698]	1.371	[.673, 2.791]	.862	[.439, 1.692]	.598	[.251, 1.425]	1.054	[.494, 2.246]
Hispanic/other	1.549	[.411, 5.833]	1.116	[.478, 2.604]	.966	[.180, 5.194]	2.012†	[.921, 4.390]	.560†	[.284, 1.102]	.701	[.320, 1.535]	.405*	[.172, .955]
< Bachelor’s degree^c^	2.864	[.798, 10.285]	1.966†	[.925, 4.181]	1.528	[.380, 6.154]	1.289	[.701, 2.369]	.773	[.439, 1.362]	.747	[.377, 1.479]	.507*	[.257, 1.000]
< Full-time employed^d^	4.795	[.604, 38.040]	1.475	[.595, 3.655]	2.956	[.343, 25.456]	1.036	[.502, 2.135]	.953	[.481, 1.887]	3.611**	[1.531, 8.520]	2.109†	[.886, 5.024]
Census region^e^														
Midwest	1.079	[.265, 4.392]	1.680	[.654, 4.310]	1.659	[.184, 14.910]	.803	[.375, 1.717]	1.631	[.750, 3.549]	.895	[.368, 2.180]	.807	[.335, 1.940]
Northeast	.513	[.185, 1.419]	1.417	[.649, 3.100]	.603	[.168, 2.160]	.677	[.346, 1.323]	1.377	[.707, 2.683]	.518	[.224, 1.200]	.632	[.297, 1.348]
West	1.176	[.337, 4.101]	1.737	[.767, 3.935]	3.035	[.345, 26.685]	1.072	[.535, 2.147]	1.495	[.777, 2.878]	1.250	[.579, 2.699]	1.146	[.532, 2.466]
Self-rated health	1.003	[.980, 1.026]	.993	[.977, 1.010]	1.015	[.985, 1.046]	1.000	[.986, 1.014]	.992	[.978, 1.006]	1.010	[.994, 1.027]	1.015†	[.998, 1.031]
Constant	12.369†	[.738, 207.190]	7.915*	[1.182, 53.007]	32.789†	[.905, 1187.550]	1.415	[.286, 7.002]	5.631*	[1.178, 26.931]	.345	[.056, 2.141]	.161*	[.026, .998]
R^2^	.072		.037		.087		.021		.033		.053			

*CI* confidence interval, *OR* odds ratio, *RR* risk ratio

^a^reference = man; ^b^reference = non-Hispanic White; ^c^reference = bachelor’s degree or higher; ^d^reference = employed full time; ^e^reference = South

^†^*p* < .10, *p < .05, **p < .01, ***p < .001

### Willingness to Participate in HIV Research Without Payment

Approximately one-third (33.90%) of respondents indicated they would be willing to participate in HIV research without pay; an additional 28.33% said “it depends,” while 38.01% would not participate without payment (see Table 1). Multinomial logistic regression (see Table 2, Model 6) revealed that women were more than twice as likely to be willing to participate in HIV research studies without pay (RR = 2.045, *p* < 0.05), controlling for all other sociodemographic variables. Participants with less than a bachelor’s degree (RR = 0.507, *p* < 0.05) and those who identified as Hispanic/other race-ethnicity (RR = 0.405, *p* < 0.05) were at least half as likely to be willing to participate in HIV research studies without pay, compared to respondents with bachelor’s degrees and non-Hispanic Whites, respectively. Respondents not employed full-time (vs. full-time employed) were more than three-times more likely to respond “it depends” (RR = 3.611, *p* < 0.01) when asked whether they would be willing to participate in HIV research without pay.

### Risk Perceptions

Nearly half (45.89%) of respondents could imagine a level of risk in an HIV intervention study that was so high that no amount of payment could convince them to participate (see Table 1). Logistic regression revealed positive associations between this outcome and age (OR = 1.035, *p* < 0.05) and living in the Northeast (vs. South) census region (OR = 2.111, *p* < 0.05; see Table 3, Model 7).

**Table 3:** Logistic regression results for can imagine a risk level high enough to deter research participation (Model 7) and linear regression results for risk perceptions (Models 8–11), by sociodemographic characteristics, *N* = 292

	Model 7		Model 8		Model 9		Model 10		Model 11	
	Can imagine a risk level high enough to deter research participation		% chance of harm that would prevent research participation		% chance of death that would prevent research participation		Perceived risk if study pays $0		Perceived risk if study pays $20,000	
	OR	CI	*b*	CI	*b*	CI	*b*	CI	*b*	CI
Age	1.035*	[1.006, 1.065]	− .055	[− .457, .348]	.128	[− .319, .575]	.009	[− .033, .052]	.015	[− .024, .054]
Woman^a^	1.021	[.611, 1.706]	6.549†	[− .816, 13.914]	4.815	[− 3.365, 12.995]	.571	[− .207, 1.350]	.363	[− .354, 1.081]
Race-ethnicity^b^										
Non-Hispanic Black	1.127	[.587, 2.164]	.771	[− 8.620, 10.163]	9.201†	[− 1.230, 19.632]	1.761***	[.768, 2.754]	− .859†	[− 1.774, .056]
Hispanic/other	1.218	[.627, 2.366]	− 8.776†	[− 18.396, .754]	− 5.056	[− 16.641, 4.529]	.888†	[− .119, 1.895]	− .390	[− 1.319, .538]
< Bachelor’s degree^c^	1.246	[.716, 2.167]	− 4.022	[− 11.991, 3.947]	− 4.676	[− 13.527, 4.174]	.413	[− .430, 1.255]	− .307	[− 1.083, .470]
< Full-time employed^d^	1.099	[.564, 2.138]	− .511	[− 10.045, 9.023]	− .878	[− 11.468, 9.711]	.329	[− .679, 1.336]	.304	[− .624, 1.233]
Census region^*e*^										
Midwest	.824	[.395, 1.717]	− .134	[− 10.569, 10.301]	.994	[− 10.596, 12.583]	− .036	[− 1.139. 1.067]	.801	[− .216, 1.818]
Northeast	2.111*	[1.104, 4.036]	− 9.581*	[− 18.857, − ﻿.305]	− 8.839†	[− 19.142, 1.464]	.376	[− .605, 1.356]	.292	[− .611, 1.196]
West	1.035	[.555, 1.933]	− 8.654†	[− 17.644, .336]	− 6.976	[− 16.961, 3.009]	.966*	[.016, 1.916]	− .182	[− 1.059, .693]
Self-rated health	1.006	[.993, 1.019]	.078	[− .112, .268]	− .000	[− .211, .210]	− .006	[− .026, .014]	.001	[− .018, .019]
Constant	.133**	[.029, 1.613]	46.129***	[24.879, 67.380]	28.711*	[5.108, 52.314]	1.830	[− .416, 4.076]	5.836***	[3.766, 7.907]
R^2^	.035		.054		.044		.079		.037	

*b* coefficient, *CI* confidence interval, *OR* odds ratio

^a^Reference = man; ^b^reference = non-Hispanic White; ^c^reference = bachelor’s degree or higher; ^d^reference = employed full-time; ^e^reference = South

^†^*p* < .10, *p < .05, **p < .01, ***p < .001

On average, respondents reported that a 45.16% chance of harm and 30.66% chance of death would prevent them from participating in HIV research (see Table 1). Multivariate linear regression analyses showed a negative correlation between chance of harm that would deter participation and living in the Northeast (vs. South) census region (*b* = -9.581, *p* < 0.05), but there were no statistically significant correlations between any sociodemographic characteristics and percent chance of death that would deter participation (see Table 3, Models 8–9).

Respondents assumed higher-paid studies were riskier than lower-paid studies, rating the mean risk of a study that paid $0 as 2.86 out of 10 and mean risk of a study that paid $20,000 as 6.40 out of 10 (see Table 1). This difference in perceived risk based on pay (3.54) was statistically significant (*t* (291) = 12.15, *p* < 0.001). Studies paying $0 were perceived to be riskier by non-Hispanic Blacks (vs. non-Hispanic Whites; *b* = 1.761, *p* < 0.001) and individuals living in the West (vs. South) census region (*b* = 0.966, *p* < 0.05). Significant sociodemographic differences in risk perceptions were not identified when studies paid $20,000 (see Table 3, Models 10–11).

### Importance of Various Forms of Payment

Respondents rated compensation for time as the most important form of payment for research participation (mean = 7.82 out of 10; see Table 1), followed by cash incentives (mean = 7.42), post-trial access to the intervention if successful (mean = 6.78), and reimbursement for lost wages (mean = 6.55). Transportation vouchers (mean = 4.78), food (mean = 4.22), and gifts (mean = 2.77) were ranked less important among possible types of payment for research participation.

Multivariate linear regression models examining sociodemographic differences in the importance of payment type (see Table 4, Models 12–18) revealed the importance of cash incentives was negatively correlated with age (*b* = -0.041, *p* < 0.05) and being a woman (vs. man; *b* = -0.868, *p* < 0.05). Reimbursement for lost wages was more important for respondents without a bachelor’s degree (vs. bachelor’s degree or higher; *b* = 1.410, *p* < 0.05) and less important for respondents not employed full-time (vs. employed full-time; *b* = -1.080, *p* < 0.05). The importance of compensation for time was negatively correlated with age (*b* = -0.043; *p* < 0.01). Transportation vouchers were more important for respondents living in the Northeast (*b* = 1.431, *p* < 0.01) and West (*b* = 1.137, *p* < 0.05) census regions versus South. Food was more important for women (vs. men; *b* = 1.014, *p* < 0.01) and respondents living in the Midwest (*b* = 1.153, *p* < 0.05) and West (*b* = 1.075, *p* < 0.05) versus South, but less important for respondents who identified as Hispanic/other race-ethnicity (*b* = -1.204, *p* < 0.05) versus non-Hispanic White. The perceived importance of gifts was negatively correlated with age (*b* = -0.046, *p* < 0.05), while the perceived importance of post-trial access to the intervention did not significantly vary by any sociodemographic characteristics.

**Table 4:** Linear regression results for importance of various forms of payment by sociodemographic characteristics, *N* = 292

	Model 12		Model 13		Model 14		Model 15		Model 16		Model 17		Model 18	
	Cash incentive		Reimbursement for lost wages		Compensation for time		Transportation voucher		Food		Gifts		Post-trial access to intervention	
	*b*	﻿CI	*b*	CI	*b*	﻿CI	*b*	﻿CI	*b*	﻿CI	*b*	CI	*b*	CI
Age	− .041*	[− .080,− ﻿.001]	− .013	[− .056, .031]	− .043**	[− .077, −﻿ .009]	− .014	[− .059, .032]	− .038†	[− .081, .006]	− .046*	[− .088, −﻿ .005]	.043†	[− .001, .086]
Woman^a^	− .868*	[− 1.591, −﻿ .145]	− .310	[− 1.108, .487]	− .436	[− 1.057, .184]	.220	[− .613, 1.053]	1.014**	[.217, 1.811]	.233	[− .529, .994]	.437	[− .352, 1.226]
Race-ethnicity^b^														
Non-Hispanic Black	− .095	[− 1.017, .827]	.111	[− .906, 1.129]	.283	[− .508, 1.074]	.479	[− .583, 1.541]	− .030	[− 1.046, .986]	.850†	[− .121, 1.821]	− .739	[− 1.746, .267]
Hispanic/other	.671	[− .265, 1.607]	− .110	[− 1.142, .922]	.350	[− .452, 1.153]	− .104	[− 1.182, .973]	− 1.204*	[− 2.235, − ﻿.173]	.517	[− .469, 1.502]	− .527	[− 1.548, .494]
< Bachelor’s degree^c^	.337	[− .446, 1.119]	1.410***	[.547, 2.273]	.064	[− .607, .735]	.876†	[− .025, 1.777]	.382	[− .480, 1.245]	− .377	[− 1.201, .447]	.192	[− .662, 1.046]
< Full-time employed^d^	.513	[− .423, 1.449]	− 1.080*	[− 2.113, −﻿ .047]	− .067	[− .870, .736]	− .394	[− 1.472, .684]	− .180	[− 1.212, .851]	− .247	[− 1.233, .739]	− .134	[− 1.156, .888]
Census region^*e*^														
Midwest	.583	[− .441, 1.608]	.391	[− .739, 1.522]	.260	[− .619, 1.139]	.931	[− .249, 2.111]	1.153*	[.024, 2.282]	.811	[− .268, 1.890]	.688	[− .430, 1.806]
Northeast	.318	[− .593, 1.228]	.162	[− .843, 1.167]	.409	[− .373, 1.190]	1.431**	[.382, 2.480]	.388	[− .616, 1.391]	.137	[− .823, 1.096]	.035	[− .959, 1.029]
West	− .085	[− .967, .798]	.725	[− .248, 1.699]	.509	[− .249, 1.266]	1.137*	[.120, 2.154]	1.075*	[.103, 2.048]	.729	[− .201, 1.658]	.174	[− .789, 1.138]
Self-rated health	.006	[− .013, .025]	.009	[− .012, .029]	.011	[− .005, .027]	.004	[− .018, .025]	− .009	[− .029, .012]	.003	[− .017, .022]	.017†	[− .003, .039]
Constant	8.327***	[6.240, 10.413]	6.055***	[3.752, 8.357]	8.346***	[6.556, 10.136]	4.003***	[1.599, 6.406]	5.398***	[3.100, 7.697]	3.693***	[1.496, 5.890]	4.064***	[1.786, 6.341]
R^2^	.049		.053		.052		.046		.071		.058		.046	

*b* coefficient, *CI* confidence interval

^a^reference = man; ^b^reference = non-Hispanic White; ^c^reference = bachelor’s degree or higher; ^d^reference = employed full-time; ^e^reference = South

^†^*p* < .10, *p < .05, **p < .01, ***p < .001

## Discussion

In this study, we aimed to understand prospective HIV research study participants’ attitudes toward payment for research participation, perceptions of the relationship between payment amount and study risk, and preferences for various forms of payment. We also sought to identify whether these attitudes, perceptions, and preferences varied by key sociodemographic characteristics. We found payment often plays a role in decision-making about HIV research participation and may shape willingness to participate in research studies differently based on an individual’s sociodemographic background. Women were more likely to be willing to participate in HIV research without payment; conversely, individuals without a bachelor’s degree and individuals identifying as being Hispanic or from another (non-Black) minoritized racial-ethnic group were less likely to be willing to participate in HIV research without payment. Previous research, including of women recruited via MTurk, has shown that women are, in general, more altruistic than men [20, 32, 33]; our finding may extend that altruism to HIV research study participation without payment. Our interpretation of lower levels of interest in HIV research participation without pay among some minoritized racial and ethnic groups and persons with less formal education is that the lack of payment acts as a disincentive to participation, perhaps due to the study risks, a lack of trust in research, or because these groups are more likely to experience income insecurity and may lack the time to participate in activities that do not provide payment as compensation [17, 34]. This corroborates our corollary finding that participants identified compensation for time as the most important form of payment for determining research participation.

That the importance of cash incentives and compensation for time were negatively correlated with age underscores how payment is viewed as *compensation*. Younger people appear to place a premium on their time and refrain from activities that do not provide something immediately beneficial, like tangible cash payments, in return [35]. This provides evidence that HIV researchers cannot rely on altruism alone to recruit diverse participant samples. Our identification of sociodemographic variation in preferences for the form of payment (e.g., cash, reimbursement for lost wages, transportation, food, gifts) by age, gender, education, race and ethnicity, and census region of residence, further suggests a one-size-fits-all approach to payment could differentially influence participation among distinct sociodemographic groups. To encourage more equitable participation in HIV research, we thus encourage institutions to commit to breaking down barriers around various forms of payment and urge researchers to ask participants what form of payment they prefer to receive, rather than choosing a form of payment on participants' behalf.

Another key finding was that respondents perceived increased study risk when offered high payment, versus no payment, for HIV research participation, illustrating how payment may influence prospective HIV research participants’ risk–benefit calculus [36]. Though perhaps unsurprising, as it is human nature to maximize benefits and reduce risk [37], IRBs explicitly prohibit the use of research incentives as a study benefit [38]. Thus, while payment may be presented as a tool to boost study recruitment efforts [3] or reimburse for time and burdens, it may be implicitly interpreted by potential HIV research participants as a means of compensating for the riskiness of the procedures involved in the research study. This finding complements previous research that has found study payment signals risk level for adults in the general population [5] and that study participants expect to be compensated for physical risks incurred [17].

An additional finding was that non-Hispanic Black respondents had elevated perceptions of risk (compared to non-Hispanic Whites) when studies did not offer any payment. This is likely due to higher perceived risks of HIV research participation among Black individuals in general, driven by both (a) the historical targeted exploitation of African Americans in health research that has led to high levels of medical mistrust in doctors and researchers today, and (b) elevated levels of HIV-related stigma in some African American communities that would make any breaches of participant confidentiality socially costly [39, 40]. Finally, regional variation existed in both perceptions of risk based on payment level and tolerance for the risk of potential harm incurred by research participation. Such variation may be driven by regional differences in several factors, including poverty, knowledge about research, trust in science, proximity to research centers, and previous experience with research participation [41, 42]. Further research is warranted regarding how perceptions of clinical, financial, social, and psychological risks in research participation may vary across geographic region and racial-ethnic groups.

### Limitations

This study is not without limitations. While we were able to recruit a U.S. national sample of people living with HIV, our sample may not be representative of the total pool of available participants—including individuals without internet access, non-English speakers, and non-MTurk workers. Previous research suggests samples recruited via MTurk overrepresent younger, White, and male populations in the United States [43], further limiting the generalizability of our findings. There is also the issue of self-selection bias, in terms of people responding to the survey because they were interested in the topic of payment. Although we had robust filters in place to control for this, other research has shown the impact of bots and otherwise ineligible or fraudulent participants infiltrating surveys to obtain payment [29, 30, 44, 45]. We used multiple screening questions to help ensure our participants were living with HIV, however we were unable to use diagnostic testing to confirm HIV status. Hence, it is possible that some individuals not living with HIV misrepresented themselves and were able to pass the screening questions and complete the survey. Finally, asking people who participate in paid surveys if they believe they should be paid to participate in research may be misleading, as completing surveys for money is part of their everyday life.

### Conclusion

We must further explore the presence of undue inducement related to allocating higher payment values on studies with increased risk and greater chance of harm, as well as the impact of low or no payment on research participation and exploitation of the research participant community. Obtaining the perspectives of other stakeholders in the research process, such as IRB members, investigators, study sponsors, and bioethicists may shed additional light on these issues. People’s attitudes toward and perceptions of appropriate payment may vary by geographic region, gender, education level, employment status, race, and ethnicity—among other factors—all of which must be further characterized in larger studies. Having data on these attitudes even in a specific research topic area like HIV is a start to developing norms and standards around payment in research and addressing payment-related barriers to equity in research participation.

## Data Availability

Due to the nature of this research, participants of this study did not agree for their data to be shared publicly, so supporting data are not available.

## References

[1] Brown B, Galea JT, Davidson P, Khoshnood K (2016). Transparency of participant incentives in HIV research. Lancet HIV.

[2] Lee R, Cui RR, Muessig KE, Thirumurthy H, Tucker JD (2014). Incentivizing HIV/STI testing: a systematic review of the literature. AIDS Behav.

[3] Grady C (2005). Payment of clinical research subjects. J Clin Invest.

[4] Bentley JP, Thacker PG (2004). The influence of risk and monetary payment on the research participation decision making process. J Med Ethics.

[5] Cryder CE, London JA, Volpp KG, Loewenstein G (2010). Informative inducement: study payment as a signal of risk. Soc Sci Med.

[6] London AJ, Borasky Jr DA, Bhan A, Ethics working group of the HIV prevention trials network. Improving ethical review of research involving incentives for health promotion. PLoS Med. 2012;9(3):e1001193. 10.1371/journal.pmed.1001193 10.1371/journal.pmed.1001193 PMC3313933 22479154

[7] Largent E (2017). For love and money: the need to rethink benefits in HIV cure studies. J Med Ethics.

[8] Halpern SD, Chowdhury M, Bayes B (2021). Effectiveness and ethics of incentives for research participation: 2 randomized clinical trials. JAMA Intern Med.

[9] Unger JM, Vaidya R, Hershman DL, Minasian LM, Fleury ME (2019). Systematic review and meta-analysis of the magnitude of structural, clinical, and physician and patient barriers to cancer clinical trial participation. J Natl Cancer Inst.

[10] Resnik DB, McCann DJ (2015). Deception by research participants. N Engl J Med.

[11] Fisher J, McManus L, Kalbaugh J, Walker RL (2021). Phase I trial compensation: how much do healthy volunteers actually earn from clinical trial enrollment. Clin Trials.

[12] Blumenthal-Barby J, Ubel P (2021). Payment of COVID-19 challenge trials: underpayment is a bigger worry than overpayment. J Med Ethics.

[13] Gelinas L, Largent EA, Cohen IG, Kornetsky S, Bierer BE, Lynch HF (2018). A framework for ethical payment to research participants. N Engl J Med.

[14] Brown B, Galea JT, Dubé K (2018). Crucial but understudied: incentives in HIV research. Lancet HIV.

[15] Brown B, Merritt MW (2013). A global public incentive database for human subjects research. IRB.

[16] Ripley E, Macrina F, Markowitz M (2006). Paying clinical research participants: one institution’s research ethics committees’ perspective. JERHRE.

[17] Devlin A, Brownstein K, Goodwin J (2021). ‘Who is going to put their life on the line for a dollar? That’s crazy’: community perspectives of financial compensation in clinical research. J Med Ethics.

[18] Dickert N, Emanuel E, Grady G (2002). Paying research subjects: an analysis of current policies. Ann Intern Med.

[19] Rhodes R, Gligorov N, Schwab A (2013). The human microbiome: ethical, legal, and social concerns.

[20] Lobato L, Bethony J, Pereira F, Grahek S, Diemert D, Gazzinelli M (2014). Impact of gender on the decision to participate in a clinical trial: a cross-sectional study. BMC Public Health.

[21] Brown B, Marg L, Michels E (2021). Comparing payments between sociobehavioral and biomedical studies in a large research university in Southern California. J Empir Res Hum Res Ethics.

[22] Karris MY, Dubé K, Moore AA (2020). What lessons it might teach us? Community engagement in HIV research. Curr Opin HIV AIDS.

[23] Bosh KA, Hall HI, Eastham L, Daskalakis DC, Mermin JH (2021). Estimated annual number of HIV infections—United States, 1981–2019. MMWR Morb Mortal Wkly Rep.

[24] Rubin MS, Colen CG, Link BG (2010). Examination of inequalities in HIV/AIDS mortality in the United States from a fundamental cause perspective. Am J Public Health.

[25] Macklin R (2003). Bioethics, vulnerability, and protection. Bioethics.

[26] Pepperrell T, Hill A, Moorhouse M (2020). Phase 3 trials of new antiretrovirals are not representative of the global HIV epidemic. J Virus Erad.

[27] Losina E, Schackman BR, Sadownik SN (2009). Racial and sex disparities in life expectancy losses among HIV-infected persons in the United States: impact of risk behavior, late initiation, and early discontinuation of antiretroviral therapy. Clin Infect Dis.

[28] Shank DB (2016). Using crowdsourcing websites for sociological research: the case of Amazon Mechanical Turk. Am Sociol.

[29] Sheehan KB (2018). Crowdsourcing research: data collection with Amazon’s Mechanical Turk. Commun Monogr.

[30] Kramer J, Rubin A, Coster W (2014). Strategies to address participant misrepresentation for eligibility in web-based research. Int J Methods Psychiatr Res.

[31] Conrique BG, McDade-Montez E, Anderson PM (2020). Detection and prevention of data fraud in a study of community college career technical education students. Community Coll J Res Pract.

[32] Brañas-Garza P, Capraro V, Rascon-Ramirez E (2018). Gender differences in altruism on Mechanical Turk: expectations and actual behaviour. Econ Lett.

[33] Nakavachara V (2018). The economics of altruism: The old, the rich, the female. J Hum Behav Soc Environ.

[34] Clark LT, Watkins L, Piña IL (2019). Increasing diversity in clinical trials: overcoming critical barriers. Curr Prob Cardiol.

[35] Bixter MT, Rogers WA (2019). Age-related differences in delay discounting: immediate reward, reward magnitude, and social influence. J Behav Dec Making.

[36] Dubé K, Dee L, Evans D (2018). Perceptions of equipoise, risk-benefit ratios, and "otherwise healthy volunteers" in the context of early-phase HIV cure research in the United States: a qualitative inquiry. JERHRE.

[37] Homans, GC. (1961). Social behavior and its elementary forms. Harcourt, Brace, and World.

[38] Department of Health‚ Education‚ and Welfare; National Commission for the Protection of Human Subjects of Biomedical and Behavioral Research. The Belmont Report. Ethical principles and guidelines for the protection of human subjects of research. J Am Coll Dent. 2014;81(3):4–13. 25951677

[39] Alsan M, Wanamaker M, Hardeman RR (2020). The Tuskegee study of untreated syphilis: a case study in peripheral trauma with implications for health professionals. J Gen Intern Med.

[40] Kerani R, Narita M, Lipira L, Endeshaw M, Holmes KK, Golden MR (2019). Challenges in recruiting African-born, US-based participants for HIV and tuberculosis research. J Immigr Minor Health.

[41] Heumann C, Cohn SE, Krishnan S (2015). Regional variation in HIV clinical trials participation in the United States. South Med J.

[42] Corbie-Smith G, Odeneye E, Banks B, Shandor Miles M, Roman IM (2013). Development of a multilevel intervention to increase HIV clinical trial participation among rural minorities. Health Educ Behav.

[43] Nadler J, Baumgartner S, Washington M (2021). MTurk for working samples: evaluation of data quality 2014–2020. N Am J Psychol.

[44] Chmielewski M, Kucker SC (2020). An MTurk crisis? Shifts in data quality and the impact on study results. Soc Psychol Personal Sci.

[45] Griffin M, Martino RJ, LoSchiavo C, Comer-Carruthers C, Krause KD, Stults CB, Halkitis PN (2021). Ensuring survey research data integrity in the era of internet bots. Qual Quant.

